# How Convolutional Neural Networks Diagnose Plant Disease

**DOI:** 10.34133/2019/9237136

**Published:** 2019-03-26

**Authors:** Yosuke Toda, Fumio Okura

**Affiliations:** ^1^Japan Science and Technology Agency, 4-1-8 Honcho, Kawaguchi, Saitama 332-0012, Japan; ^2^Institute of Transformative Bio-Molecules (WPI-ITbM), Nagoya University, Chikusa, Nagoya 464-8602, Japan; ^3^Department of Intelligent Media, Institute of Scientific and Industrial Research, Osaka University, 8-1 Mihogaoka, Ibaraki, Osaka 567-0047, Japan

## Abstract

Deep learning with convolutional neural networks (CNNs) has achieved great success in the classification of various plant diseases. However, a limited number of studies have elucidated the process of inference, leaving it as an untouchable* black box*. Revealing the CNN to extract the learned feature as an interpretable form not only ensures its reliability but also enables the validation of the model authenticity and the training dataset by human intervention. In this study, a variety of neuron-wise and layer-wise visualization methods were applied using a CNN, trained with a publicly available plant disease image dataset. We showed that neural networks can capture the colors and textures of lesions specific to respective diseases upon diagnosis, which resembles human decision-making. While several visualization methods were used as they are, others had to be optimized to target a specific layer that fully captures the features to generate consequential outputs. Moreover, by interpreting the generated attention maps, we identified several layers that were not contributing to inference and removed such layers inside the network, decreasing the number of parameters by 75% without affecting the classification accuracy. The results provide an impetus for the CNN* black box* users in the field of plant science to better understand the diagnosis process and lead to further efficient use of deep learning for plant disease diagnosis.

## 1. Introduction

Plant disease has long been one of the major threats to food security because it dramatically reduces the crop yield and compromises its quality. Accurate and precise diagnosis of diseases has been a significant challenge. Traditionally, identification of plant diseases has relied on human annotation by visual inspection. Nowadays, it is combined or substituted with various technologies such as immunoassays (e.g., enzyme-linked immunosorbent assay, ELISA) and PCR or RNA-seq to detect pathogen-specific antigens or oligonucleotides, respectively [1, 2]. Moreover, recent technical advances and dramatic cost reductions in the field of digital image acquisition have allowed the introduction of an array of image-based diagnosis methods at a practical level [3]. However, as the acquired image encloses condensed information that is extremely difficult for the computer to process, it requires a preprocessing step to extract a certain feature (e.g., color and shape) that is manually predefined by experts [4, 5]. In such situations, deep learning is typically used because it allows the computer to autonomously learn the most suitable feature without human intervention. An initial attempt to use deep learning for image-based plant disease diagnosis was reported in 2016, where the trained model was able to classify 14 crops and 26 diseases with an accuracy of 99.35% against optical images [6]. Since then, successive generations of deep-learning-based disease diagnosis in various crops have been reported [7–13].

Among various network architectures used in deep learning, convolutional neural networks (CNN) are widely used in image recognition. The first CNNs, the neocognitron [14] and LeNet [15], were introduced in the 1980s, although the study of neural networks originally started in the 1940s [16]. CNNs have been used for plant image analysis since the early days of their evolution [17]. Thanks to the rapid development of hardware and the improvement of learning methods [18], large-scale deep CNNs became trainable in the 2010s. A major turning point for the CNNs was the introduction of AlexNet [19], which significantly outperformed the image classification accuracy of traditional machine learning approaches in ImageNet Large Scale Visual Recognition Challenge (LSVRC) 2012 [20].

CNNs consist of convolutional layers, which are sets of image filters convoluted to images or feature maps, along with other (e.g., pooling) layers. In image classification, feature maps are extracted through convolution and other processing layers repetitively and the network eventually outputs a label indicating an estimated class. Given a training dataset, CNN, unlike traditional machine learning techniques that use* hand-crafted* features [21], optimizes the weights and filter parameters in the hidden layers to generate features suitable to solve the classification problem. In principle, the parameters in the network are optimized by back-propagation [22] and gradient descent approaches [23] to minimize the classification error.

After the invention of AlexNet, along with the advances in hardware, the CNN architecture became larger. VGG-19 consists of 19 layers [24], while GoogLeNet [25] has 22 layers with junctions in its architecture. In LSVRC 2015, ResNet [26] outperformed the classification accuracy of the human-level performance with a 152-layer network. However, complexity of the CNN architecture, which generally contributes to higher accuracy, has caused significant problems for interpretability and raised the following questions: What does CNN actually do in hidden layers? What feature in the input image contributes to inference and why the CNN diagnoses a specific disease? How can we validate the model if we do not know what type of data is processed inside? Deep learning was regarded as a* “black box”* [27], which prevented the use of CNNs in practical applications. Moreover, the European Union's new General Data Protection Regulation (GDPR) raises a potential concern for CNN deployment without conferring interpretability (https://gdpr-info.eu/art-22-gdpr/). Similarly, in Japan, “Draft AI R&D Guidelines for International Discussions” published by the Ministry of Internal Affairs and Communications in 2017 state that developers of artificial intelligence should make best endeavors for its accountability (http://www.soumu.go.jp/main_content/000507517.pdf). Thus, revealing the approaches that describe the network has become crucial.

The contents of the* black box* are being unveiled owing to the recent growth of the deep learning research. Researchers attempt to understand CNNs by extracting their calculation process in a human-interpretable form, such as by visualization. In the early times, Zeiler and Fergus visualized the activation at intermediate layers [28]. Several studies synthesized the images that maximize the activation to visualize features frequently used to make decisions [29, 30]. A major approach is to visualize the region important for classification within the input image such as using deconvolution [28, 31], class activation mapping (CAM) [32], or guided back-propagation [33–35]. These methods have been successful in locating the objects within the image as well as actualizing important features. However, they were often established based on the CNN trained using ImageNet, which consists of images from a large variety of objects (1,000 categories). In contrast, datasets of plant diseases differ from others by both the variation and size of the features required for classification. It is axiomatic that disease diagnosis cannot be equated to classify cats and dogs because the former relies on subtle differences (e.g., lesions that appear on the leaf) compared to the latter. Researchers have applied the visualization methods to extract the representation of plant diseases from trained CNNs ([9, 36, 37]). Comparisons of these visualization methods have also been performed [38]. A novel visualization method to detect a lesion caused by a plant disease has been recently proposed [39].

In this study, based on the findings of the previous studies, we provide a deeper evaluation of the visualization methods against the CNNs in plant science applications. Our results show that several visualization methods are usable in their original form, indicating that the CNN captures the lesion-specific features of respective diseases. However, several methods have to go through a process of targeted layer optimization to generate an optimum result owing to the differences in the CNN architecture and the datasets. Moreover, based on the layer-wise visualization, we identify an optimal number of feature extraction layers to simplify the CNNs by decreasing the number of network parameters by 75%.

*Contributions. *The following are the contributions of this study. First, this study is the first attempt of comprehensive analyses which studies what the CNNs learn during the plant disease diagnosis. This is a significant problem for the rapid development of deep learning techniques in the plant phenotyping tasks. It constructs a standard for selecting and interpreting CNN models for plant image analysis. Second, from the computer science perspective, this study provides novel results by the visualization of a CNN applied for plant image analysis. The trend of the visualizations is notably different from previous discussions in visualization analyses for general object recognition.

## 2. Materials and Methods

### 2.1. Experimental and Technical Design

To unveil the characteristics of visualization approaches for CNNs for plant disease diagnosis, we adopted various methods on a trained CNN model using a leaf disease dataset. We compared four categories of visualization methods, (I) hidden layer output visualization [6], (II) feature visualization [40, 41], (III) semantic dictionary [42], and (IV) attention map [28, 29, 33, 34, 43–45]. Representative images generated by the respective methods are described in Figure S1. Although some methods suggest specific layer settings for visualization (e.g., visualizing the first layer produced better results), we visualized each layer to investigate the behavior of the methods in practical settings for plant disease diagnosis.

### 2.2. Dataset and Network for Disease Diagnosis Training

**Figure 1:**
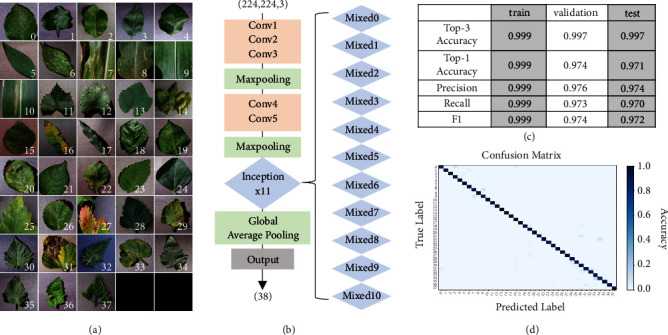
*Image-based disease diagnosis training using convolutional neural networks.* (a) The PlantVillage image dataset used in this study. This dataset contains 38 categories of diseased or healthy leaf images. See Figure S2 for the names of species and diseases assigned to each label. (b) InceptionV3-based convolutional neural network (CNN) architecture used in this study. Conv, convolutional layer; Mixed, inception module. (c) Accuracy, precision, recall, and mean F1 scores against the training, validation, and test data using the trained weights. (d) Confusion matrix drawn against the test dataset. See Figure S2 for an enlarged view.

Images used in this report were adopted from the PlantVillage dataset [46] (https://github.com/spMohanty/PlantVillage-Dataset). This dataset comprises healthy or diseased leaf images classified into 38 labels (54,306 images, 26 diseases, 14 crop species) (Figure 1(a)). Images were split into training, validation, and test datasets with a ratio of 6:2:2. Using such images, we prepared a CNN based on InceptionV3 [47] which receives a three-channel input image of 224 x 224 resolution and returns a 38-dimensional vector (Figure 1(b)). We selected this network architecture because it is comprised of repeating convolution blocks without complex layers such as residual connections [26] that will make the interpretation of the intermediate layers difficult. Network weights with the lowest validation loss (16th epoch) were used for the test phase. The accuracy and loss values of the training, validation, and test datasets are summarized in Figure 1(c). The confusion matrix indicates that there is no imbalanced accuracy in any class (Figure 1(d) and Figure S2). We used this set of weights to interpret how the neural network has learned to diagnose the plant disease.

Training of CNN was performed using a Python library called Keras with Tensorflow backend [48], which is a deep learning framework. Pixel values of input images were divided by 255 so that they range within [0.0,1.0]. The network was initialized with random weights. Using a categorical cross-entropy loss metric, network weights were optimized using the Adam optimization algorithm with a learning rate of 0.05. A set of 128 images with a size of 224 x 224 were fed to the network as a batch per iteration. It required 3 to 4 minutes per epoch in our experimental condition (single GPU; NVIDIA GTX 1080ti). After a successful training of the CNN, the feature extraction layers (from Conv1 to global-average-pooling layer, Figure 1(b)) were optimized to capture specific features from the image for the diagnosis of the plant disease.

### 2.3. Visualization I: Hidden Layer Output Visualization

We first used one of the most naïve ways to visualize the learned features and to extract the hidden layer output (i.e., intermediate output); we passed an image to the CNN and halted the calculation at the layer of interest [6]. Since a feature extraction layer passes only the positive values to the proceeding layer because our network applies the rectified linear unit (ReLU) activation function [49], simply visualizing the intermediate outputs can provide a rough implementation of “What part of the image was important for the inference?”. As for the implementation, the related work [6] specifically focused on the output of the first convolutional layer, while we employed the same technique for each layer output.

### 2.4. Visualization II: Feature Visualization

“Feature visualization”, initially named “activation maximization” [40], was used to visualize the features that the CNN has learned by observing the activation of respective neurons with a gradient ascent-based approach. In this method, we feed a random-noise image to the neural network and calculate the gradient of the input image with respect to the mean output values of the neuron of interest. By repetitively adding the gradients to the input image, we can optimize the image to the direction that the neuron highly activates for the visualization of the feature that the neuron captures.

Since the network architecture we used is not identical to that in the original work, which used GoogLeNet [41], to investigate the effect of both differences in the network architecture and the dataset, we compared the visualizations using the CNN trained with the ImageNet dataset [20] (used in the original work) and the PlantVillage dataset. The only difference from our network is that the output layer contains 1,000 neurons instead of 38, corresponding to the number of ImageNet categories.

For feature visualization, we customized the part of the codes of the Lucid library (https://github.com/tensorflow/lucid) so that the CNN models trained by Keras can be directly used (see Code Availability). Default settings of Lucid were used for image generation. Noised images for initial input data were drawn in a color-decorrelated Fourier-transformed space. The images were fed to the CNN and the mean output values of the neuron of interest were obtained. The gradient of the input with respect to the neuron output was calculated and gradient ascent optimization was performed against the neuron of interest by Adam optimizer with a learning rate of 0.05. No regularizations were considered upon iteration. To evaluate the complexity of the features, Shannon entropy was calculated by first converting the visualized images to grayscale and using the* shannon_entropy* module of scikit-image library.

### 2.5. Visualization III: Semantic Dictionary

“Semantic dictionary” [42] is a method that combines feature visualization and intermediate output visualization and enables the better understanding of the process of diagnosis. While the previous report focuses on the intermediate output values of the convolutional layers and intends to apply feature visualization against groups of neurons, we create a neuron-wise semantic dictionary in the global average pooling (GAP) layer. The pre-softmax score of respective diseases in the output layer is calculated by the dot product of the GAP output (2048 dimensions) and the weights, which connect the GAP layer and the 38-dimensional output, added by biases. Since no further calculation is performed except for the softmax normalization to compute the output values, we can define the individual values prior to summation as a contribution score of the GAP output neurons per disease. In other words, semantic dictionary in the GAP not just allows the identification of highly contributing neurons for inference but also visualizes which type of feature was important by applying feature visualization to each neuron. To compute the contribution scores of neurons, we fed the pretrained CNN with the images of a specific disease (e.g., tomato early blight) from the test dataset and calculated the average contribution score of neurons. We observed the semantic dictionary associated with the highly contributing neurons for the diagnosis of a specific class.

### 2.6. Visualization IV: Attention Map

We generated attention maps to obtain spatial information within the input image that supports the inference as visual interpretable hotspots. For the generation of attention maps, we selected images from three categories (corn northern leaf blight-CNLB, potato early blight-PEB, and strawberry leaf scorch-SLS), where each disease displays distinct patterns of lesions (e.g., number, size, and color). These categories were often used to evaluate various visualization methods. Many approaches have been proposed to generate attention maps and we compared the following state-of-the-art representatives:

*(IV-A) Perturbation-Based Visualization. *

- Occlusion analysis [28]
- Local interpretable model-agnostic explanations (LIME) [43]

*(IV-B) Gradient-Based Visualization. *

- Vanilla back-propagation [29]
- Integrated gradients [44]
- Guided back-propagation [33]
- Grad-CAM [34]

*(IV-C) Reference-Based Visualization. *

- DeepLIFT [45]
- Explanation map [39]

Occlusion analysis [28] visualizes the degree of contribution of the masked region (or the unmasked regions) upon inference by masking a part of the input image and evaluating how the result of inference was affected compared to that of an unmodified image. Since the input images are perturbed upon analysis, such methods are classified as perturbation-based visualization approaches. Specifically, the method that highlights important regions by creating a series of perturbed images by sliding a fixed size of a mask through the images is called occlusion analysis. LIME [43] is an extension of occlusion analysis, where perturbed images are created by a combination of contiguous super pixels generated by region segmentation, followed by linear regression to obtain the contributing weights of respective super pixels against the inference.

In gradient-based visualization approaches, the gradient of the inference with respect to the input image is used to obtain the spatial information of the input, initially called a saliency map (here, vanilla back-propagation) [29]. Since then, modified methods have been proposed to improve the specificity for detecting distinctive features within an image. These include the integrated gradients method [44], which involves the computation of multiple vanilla back-propagations for images that range from black to the original input and cumulate the results and the guided ReLU-based back-propagation method [33], which is a combination of vanilla back-propagation and Deconvnet [28]. While these methods utilize the gradient of the input image, Grad-CAM [34] uses the gradient of the final layer output in the CNN which holds spatial information. Using it as a weight, the localization map is synthesized from the weighted sum of the intermediate outputs.

Reference-based visualizations were proposed based on the concept of introducing “scientific control” to the visualizations. Upon generating the attention map using the input image, additional data that serve as a reference to the input image are also fed to the network for normalization. In the case of a plant disease, the reference data that corresponds to the diseased leaf image is a healthy leaf image of the same species. DeepLIFT [45] is a method that back-propagates “contribution scores” instead of gradients; the former are calculated by using the relative activation values of neurons compared to those of the reference data. Explanation map can handle batches of reference images for normalization by first calculating the mean activation value of the respective neurons when the reference images are fed and then defining an “activation threshold” for normalization. Instead of using the gradients, the activation thresholds are used for normalizing the intermediate outputs and the sum of the top three highly activated outputs is used for attention map generation. In the original report [39], the authors claim that applying the explanation map to the first (i.e., shallowest) convolution layer with healthy leaf images as a reference can specifically highlight the lesions within the image. Notably, the major difference between DeepLIFT and the explanation map is that the former is calculated using values obtained by back-propagation, while the latter is calculated by the values obtained only through forward propagation.

We implemented the attention map visualization methods (occlusion, vanilla back-propagation, guided back-propagation, integrated gradients, Grad-CAM, and explanation map) so that they can be run with the model built in Keras. The only exception was DeepLift (with rescale rule) [45], where we employed the implementation in the DeepExplain (https://github.com/marcoancona/DeepExplain) library.

### 2.7. Code Availability

Codes required for feature visualization and attention maps are available at the following GitHub repositories: https://github.com/totti0223/lucid4keras, https://github.com/totti0223/keraswhitebox.

## 3. Results

### 3.1. Visualization I: Hidden Layer Output Visualization

**Figure 2:**
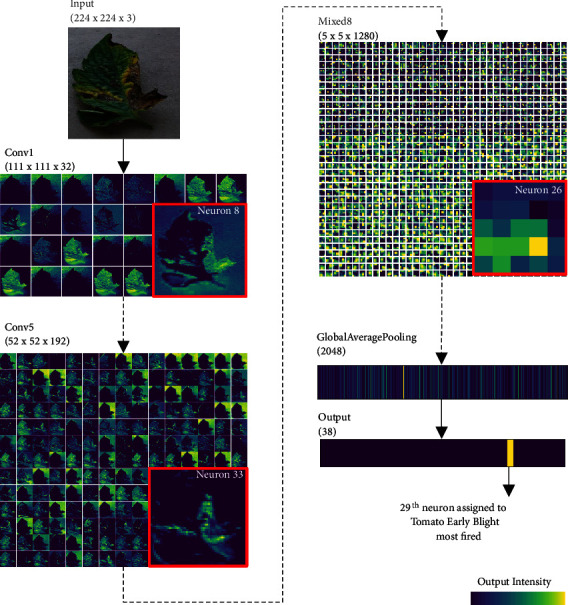
*Visualization of intermediate outputs generated by the trained CNN*. Image of tomato leaf infected with early blight (label 29) was fed to the network and intermediate output values of representative layers were visualized. Layer or inception module names and their output array sizes are described above each intermediate output.

Figure 2 visualizes the hidden layer output [6] for each layer, where an input image of tomato early blight and its generated intermediate outputs are summarized. In our trained model, some of the intermediate outputs in the shallow layers (Conv1, Conv5) highlight the yellow and brown lesions that are apparent within the image (insets with red border). However, in the deeper layer (Mixed8), owing to the convolution and pooling (i.e., downsample) layers, the image size is too small to interpret whether such extracted features have been retained. Moreover, the global average pooling layer converts images to a feature vector that eliminates the spatial information, making it highly difficult to understand how the features are handled in proceeding layers. It is difficult to distinguish whether the extracted features positively contribute to the classification of the input image to the correct disease class or are used for a reason to deny other possibilities (e.g., a furry tail raises a possibility of an image containing a cat or a dog but certainly not a car). Hence, understanding what the CNN has learned by only exploring the intermediate output is insufficient.

### 3.2. Visualization II: Feature Visualization

**Figure 3:**
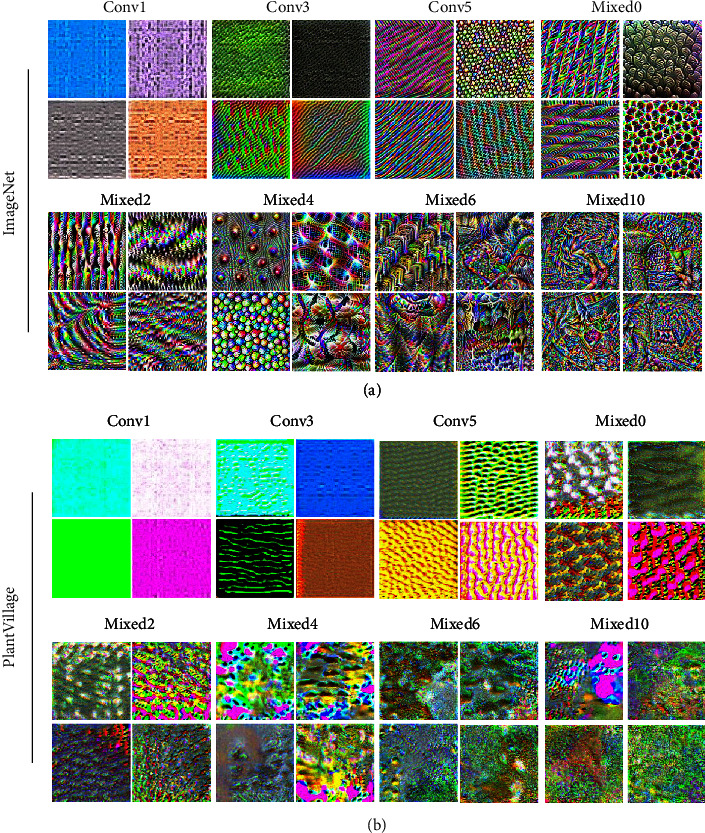
*Feature visualization*. Four neurons were randomly selected from the indicated layers and feature visualization was performed to obtain a visual interpretation of what the neurons have learned. Neurons trained with (a) ImageNet or (b) PlantVillage were, respectively, visualized.

Visualizations by the feature visualization method [40] applied on ImageNet and PlantVillage datasets are shown in Figure 3. For the ImageNet dataset, when feature visualization was applied to neurons in the shallow layers (Conv1, Conv3, and Conv5), images containing simple patterns and textures were generated. In deeper layers (Mixed0, Mixed2, and Mixed4), both the complexity of the shape and the diversity of colors increased, forming various regular patterns or object-like appearances. In even deeper layers (Mixed 6, Mixed10), several objects were intermixed, resulting in an appearance similar to abstract paintings. The increasing Shannon-entropy values [50] of the images in proportion to the depth of the network also suggest the increasing complexity (Figure S3). Thus, feature visualization can highlight the hierarchical features of what the CNN has learned. Since similar results were previously reported using the same method against neurons of the ImageNet-trained GoogLeNet [41], we confirmed that the ability of the CNN is invariant from its architecture.

For the PlantVillage dataset (Figure 3(a), right), similar to the ImageNet-trained network, neurons in the shallow layer generated simple textures (Conv1 to Conv5). Although the complexity of the image increased, it favored an edgeless abstract pattern comprised of a limited number of colors, in contrast to that of ImageNet-trained neurons. Since PlantVillage is a dataset whose images consist of a single leaf in a uniform background, learning only the green, yellow, and brown colors may have been sufficient to describe the features of the leaves and their lesions, while pink and blue are considered background colors. Moreover, the overall edgeless and obscure images resemble the visual cues of the lesions. Foliar symptoms of lesions caused by pathogens are characterized by their colors and textures rather than shapes and sizes because the shapes and sizes are often indeterminate, and feature learning of plant diseases is possibly prioritized by the colors and textures. Collectively, feature visualization can provide an implementation of the lesion features that the neurons of the CNN have learnt. However, it is unknown if the neuron has an important role upon inference. Combination with the input data, such as semantic dictionary described below, will allow further interpretability of the network.

### 3.3. Visualization III: Semantic Dictionary

**Figure 4:**
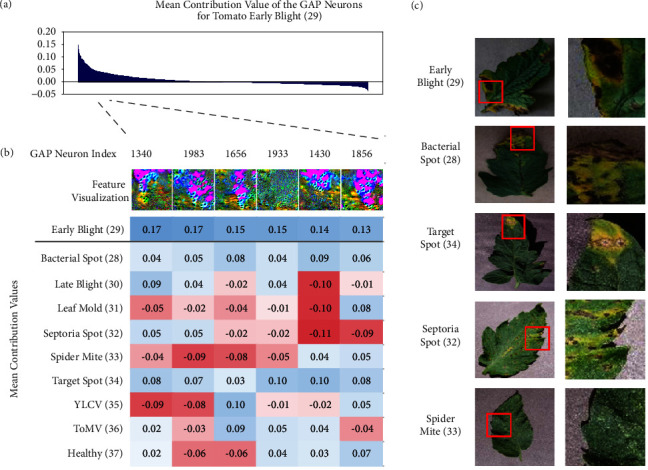
*Semantic dictionary*. Semantic dictionary generated using the intermediate outputs of global average pooling (GAP) layer. (a) Mean GAP output of 200 images of tomato early blight from the test dataset was multiplied by the weights of the CNN and sorted based on its value. (b) Neurons that correspond to the top five output values were selected and feature visualization was, respectively, applied. (c) Representative images of disease symptoms selected from the indicated class. Bottom row is a magnified view of the red inset in the top row.

Figure 4 illustrates the visualization for the highly contributed neurons (Neuron index: 1340, 1983, 1656, 1933, 1430, and 1856) in the global average pooling (GAP) layer and their contribution scores generated by semantic dictionary [42] for 200 images of tomato early blight (see Materials and Methods for details of contribution score calculation). We also show the contribution scores for other diseases of the tomato plant (Figure 4(b)). Feature visualization of the top six contributing neurons for early blight (label 29) displayed a mixture of yellow, green with partially brown area with a smooth purple, and blue texture (Figure 4(b), feature visualization). The former are the typical symptoms of early blight; dark colored lesions are accompanied with peripheral yellowing (Figure 4(c), red inset), implying that such features are important for diagnosis. The latter texture reflects the constituents of the background color. These neurons positively contribute to bacterial spots to a certain extent (label 28) and target spots (label 34) that display a similar phenotype to early blight (Figures 4(b) and 4(c)). However, they hardly or negatively contribute to septoria spots (label 32) and spider mite (label 33) whose lesions have subtle or no yellowing at all (Figures 4(b) and 4(c)). These results suggest that, similar to human decisions, CNNs extract a feature of a lesion from an image and specifically assign a positive score to diseases with a similar phenotype. Collectively, semantic dictionaries applied to the penultimate layer of CNN can highlight the features that are frequently used for disease diagnosis as a reasonable and interpretable information.

### 3.4. Visualization IV: Attention Map

**Figure 5:**
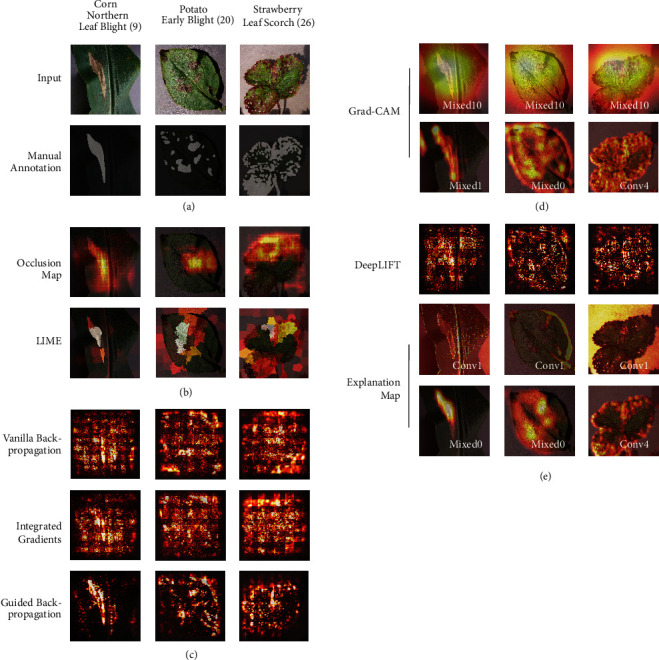
*Evaluation of attention map generating algorithms*. (a) Input images from three classes used for evaluation (top row). Numbers in parentheses indicate the class label of the dataset. Lesions in the image were manually annotated (bottom row). (b)-(e) Attention map generating methods applied to each image and displayed as a heatmap over the input. See Materials and Methods for details. (b) Perturbation-based visualization. (c) Gradient-based visualization. (d) Grad-CAM visualization. (e) Reference-based visualization. For Grad-CAM and explanation map, the layers of which the gradient and the intermediate output were used are indicated.

Using the visualization methods I–III allows neuron-wise understanding of the CNN. However, if spatial information is not considered, we cannot comprehend which part of the input image is critical upon diagnosis. Therefore, we adopted the attention map generating methods to obtain such information.  Figure 5 summarizes the visualization by such methods for three classes, CNLB, PEB, and SLS (Figure 5(a), top row). We manually annotated the lesions within each image (Figure 5(a), bottom row) and used them to evaluate the respective methods.

#### 3.4.1. Perturbation-Based Visualization

Heatmaps generated by occlusion analysis [28] are displayed in Figure 5(b). The hotspots in the heatmap overlap with the most apparent lesion in CNLB (Figure 5(b), top left panel), indicating that such regions are the most important ones for diagnosis. However, occlusion analysis fails to detect multiple lesions such as PEB and SLS (Figure 5(b), top center and top right panel) because the CNN is trained to classify the type of the disease and not its severity (e.g., by numbers, areas, or textures of the lesion) and can infer the proper class from the unmasked regions.

LIME [43] visualization partially improved the localization of lesions in PEB and SLS (Figure 5(b), bottom center and bottom right panel) where the original occlusion analysis failed (Figure 5(b) top row) by highlighting the clusters or lesions with large areas. However, LIME did not mark the relatively small or sparsely distributed lesions owing to the same reasons that occlusion analysis suffers from. Collectively, applying the perturbation-based visualization to disease classification is successful only when the size and number of lesions within the image are limited. Notably, such methods require multiple inferences to obtain a heatmap per input image, making the analysis computationally expensive and slow for real-time analysis or for mobile device deployment.

#### 3.4.2. Gradient-Based Visualization

Figures 5(c) and 5(d) show the results of the gradient-based methods. The sensitivity of guided back-propagation [33] exceeds that of vanilla back-propagation [29] and integrated gradients [44] regardless of the disease; however, the results of these methods suffer from insufficient specificity (noisy backgrounds in Figure 5(c)). Since the calculation cost of the gradients is generally lower than that of repetitive input image generation and inference required in perturbation-based methods, using guided back-propagation alone or in combination with other methods may enhance the specificity of visualization.

Applying Grad-CAM [34] with guided ReLU broadly highlighted the leaf within the image, which also lacked specificity (Figure 5(d), top row) because the resolution of the generated image is dependent on the size of the intermediate output (7 x 7 resolution for Mixed10). We applied this method with shallower layers to obtain a sharper image. Although the discriminative ability of Grad-CAM decreases upon application against shallower layers [34]; surprisingly, the shallow layers highlighted the lesions better than the deeper ones (Figure 5(d), bottom row). Overall, meaningful Grad-CAM outputs were generated from Conv2 to Mixed0 layers. In even deeper layers (i.e., deeper than Mixed5 layer), the resolution decreased, yet the location of the hotspot did not change (Figure S4). These results suggest that the weights in the shallow layers were sufficient to fully capture the features of the lesions to describe the Grad-CAM image. Using Grad-CAM is effective to create a saliency map, but, based on our results, the suitable layer must first be investigated for each application.

#### 3.4.3. Reference-Based Visualization

Figure 5(e) shows the results of the DeepLIFT [45] and explanation map [39] methods proposed to introduce “scientific control” to the visualizations. DeepLIFT improves the specificity of lesion detection compared to vanilla back-propagation and integrated gradients, but it is equivalent to or slightly superior to guided back-propagation (Figure 5(e), top row). Although we randomly selected a healthy leaf image from the same species as reference images, since the result of DeepLIFT depends on the reference, the selection of the most suitable image that represents the reference class should be carefully selected in practice.

The results of the explanation map method [39] in the original setting (i.e., applied to the shallowest layer) failed to generate a meaningful image (Figure 5(e), middle row). However, similar to the case of Grad-CAM, applying the explanation map method to the deeper layers (Mixed0 and Conv4) resulted in successful visualization (Figure 5(e), bottom row and Figure S5). While this result may be attributed to the difference in the architecture of our model, which is more complex than the network used in the original work, the difference in the dataset may also affect the visualization characteristics. Since the model in the previous report trained on a dataset of segmented leaf images with no background, the features of diseases may have been sufficiently captured in the first convolutional layer in that model. According to the visualization, our model captures the background in the early layer prior to the learning of the lesions. These results suggest that it is important to identify suitable layers for creating an effective visualization prior to applying the explanation map. Nonetheless, since this method utilizes only the intermediate output with precalculated activation thresholds without gradient computation, this is one of the most cost-efficient and explicable methods of the visualization of plant diseases.

### 3.5. Application I: Interpreting the Reasons for Misclassification by Attention Maps

**Figure 6:**
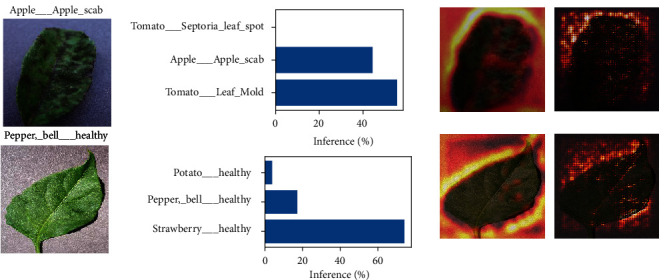
*Application of attention map generating algorithms on images misclassified by the CNN*. From left to right column: (1), images randomly selected from the dataset which were misclassified by the CNN; correct labels are displayed on top of the image; (2), the top three inferences by the CNN; (3), Grad-CAM-based visualization targeted to Mixed0 layer; (4), guided back-propagation-based visualization.

As described in the previous section, attention maps can highlight regions within the image which are important for classification. Meanwhile, applying the visualization methods on the misclassified images enables us to understand the reason why the CNN made an error.  Figure 6 shows the result of applying Grad-CAM and guided back-propagation to such images. Interestingly, both methods tended to highlight the background and contours of the leaf, instead of the leaf itself. This raises the possibility that the shape of the leaf or its background colors and textures may have been similar to that of the misclassified category. In such cases, misclassifications can be resolved by applying data augmentation to transform the shape of the leaf, introducing more images to the misclassified category so that the variation of the leaf shape will increase, or preparing background removed images upon CNN training. As described, visualization methods can reveal the potential dataset bias, which can be a basis for creating a model with higher accuracy.

### 3.6. Application II: Shaving Feature Extraction Layers from CNN

**Figure 7:**
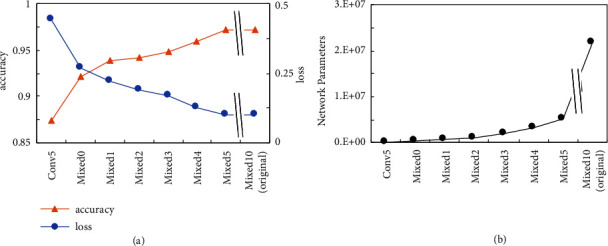
*Effect of feature extraction layer shaving*. (a) The accuracy and loss value against the test dataset of CNN whose layers posterior to the indicated layers were removed. We performed a transfer learning with the newly prepared global average pooling and output layers. Since the Mixed5 CNN showed a classification performance equivalent to the original model, further analysis was not performed. (b) Network parameters required to run the CNN.

The existence of the visualization-effective layers prior to the penultimate Mixed10 layer raises the possibility that feature extraction for diagnosis is sufficient in the shallower layers of the network. In order to verify such a possibility, we connected the shallow layers of the trained network to the GAP and the output layers (i.e., the deeper convolutional layers were removed) and performed a transfer learning (Figure 7(a)). As a result, a model whose feature extraction ended at the Mixed5 layer showed 97.14% accuracy and 0.097 loss value, which were equivalent to those of the original model (97.15% and 0.098, resp.). Increasing the number of removed layers resulted in a gradual decrease in accuracy (approximately 1% decrease for excluding one InceptionV3 module). The model that ended at Mixed5 contains only 5,167,878 parameters, while the original model has 21,880,646; that is, 75% of the parameters in the initial model can be omitted without performance degradation. When used in practical situations such as plant diagnosis with mobile devices, reducing the numbers of network parameters is important for the memory and calculation efficiency. Collectively, these results suggest that network parameters of the CNNs can be reduced by interpreting the visualization results and examining the layer contribution upon inference.

## 4. Discussion

In this study, we evaluated an array of visualization methods to interpret the representation of plant diseases that the CNN has diagnosed. The experimental results show that some simple approaches, such as naive visualization of the hidden layer output, are insufficient for plant disease visualization, whereas several state-of-the-art approaches have potential practical applications. Feature visualization and semantic dictionary can be used to extract the visual features that are heavily used to classify a particular disease. To understand what part of the input image is important, the interpretation of attention maps is a favorable choice. However, the behavior of some approaches for generating attention maps was different from what the original study suggested because previous experiments utilized the general object recognition dataset (i.e., ImageNet), which requires the extraction of fine-grained differences, unlike the plant disease diagnosis. Our task is similar to domain-specific fine-grained visual categorization (FGVC) [51], which occasionally makes the problem more challenging. This is somewhat related to the datasets of natural images (e.g., iNaturalist dataset [52]) that contain categories with a similar appearance. It is important to further understand what the deep networks learn for such fine-grained categorization tasks.

In practice, the selection of the visualization-effective layer is largely important. Even the explanation map, developed for the lesion detection of plant diseases, surprisingly shows the characteristics different from those in the original literature because of the differences in the network architecture and the dataset. Therefore, we proposed to visualize each layer and investigate which layer is most suitable for visualization.

In our experiment, the most descriptive approaches to generate layer-wise attention maps that highlight the lesions with high specificity were Grad-CAM and the explanation map (Figure 5). Notably, these are also the most cost-efficient among the evaluated methods. Grad-CAM calculates the gradient of the intermediate map with respect to the inference result and therefore requires fewer calculation steps than other gradient-based approaches that require the gradient of the input image. Moreover, the explanation map only uses intermediate output values obtained in the course of inference. Using these methods to generate attention maps for each layer is suitable for repetitive evaluation in model development or implementation in mobile devices for on-site diagnosis, as well as for benchmark analysis in the development of new attention map-generating methods for diagnosis visualization.

The comparison of visualization methods highlighted the most apparent lesions within each image. Using datasets combined with annotation labels for regions of the lesions, which are often created for semantic segmentation tasks, enables the evaluation of specificity and sensitivity of the respective methods by qualitative metrics. Nonetheless, CNN may focus on the features that we do not expect. In such cases, careful decisions on whether such features have physiological significance should be made to avoid overfitting or dataset bias.

According to the visualization results, we were able to remove 75% of the network parameters by omitting the feature extraction layers posterior to Mixed5, while not affecting the classification accuracy and the loss value (Figure 7). InceptionV3 was initially designed for training against ImageNet [47]; therefore, the shallow layers were sufficient for extracting the features required for images in PlantVillage. The visualization-based layer shaving approach is a quick and intuitive method for parameter reduction. The number of removable layers probably depends on the network architecture and the dataset the network was trained on. Training the CNN for more complex classification tasks such as plant stress (e.g., drought) may capture the features in the deeper layers of the network. Such optimal layers can be identified by the visualization methods introduced in this study.

Unlike other parameter reduction methods (pruning [53] and distillation [54]), our approach can leverage the knowledge of a specific domain (e.g., plant science) via the visualization of each layer, while the automatic methods can enable further parameter reduction. Some automatic pruning approaches utilize the amount of activation, which is often used for CNN visualization. Investigating the relationship between the existent parameter reduction approaches and the visualization methods is an interesting future direction to actualize interpretable parameter reduction of deep learning networks.

Collectively, the visualization of CNN shows the possibility to open the* black box* of deep learning. The barriers to using deep learning techniques decrease every year; however, it is important for plant scientists to select the suitable network models and interpret the outcoming results. The visualization is effective to understand what the deep network learns and it contributes to the improvement of the network architecture such as model selection and parameter reduction. Our results indicate that even if the visualization methods generate meaningful results, humans still play the most important role in evaluating the visualization results by connecting the computer-generated results with professional knowledge, for example, in plant science. Our study, which unveils the characteristics of visualization methods for disease diagnosis, opens a new path to generate a workflow for plant science studies, where computers and plant scientists cooperatively work to understand the biology of plants through machine/deep learning models.

## Data Availability

All data and codes are available upon reasonable request.

## Supplementary Materials

Supplementary Materials
* Figure S1: overview of the visualization methods introduced in this paper*. An image of Cavalier King Charles Spaniel is passed to the CNNs that were trained with ImageNet dataset. CNN predicts the image as “Blenheim_splaniel” (A breed of Cavalier) by 95.4%. Example images generated by Visualizations I to IV are displayed. See Materials and Methods for details of respective methods.* Figure S2: details of the confusion matrix described in*Figure 1(d). Ratio of classified images is described in each cell. Ticks represent the labels of the PlantVillage dataset. Class names corresponding to each label are as follows: 0, Apple___Apple_scab; 1, Apple___Black_rot; 2, Apple___Cedar_apple_rust; 3, Apple___healthy; 4, Blueberry___healthy; 5, Cherry_(including_sour)___healthy; 6, Cherry_(including_sour)___Powdery_mildew; 7, Corn_(maize)___Cercospora_leaf_spot Gray_leaf_spot; 8, Corn_(maize)___Common_rust; 9, Corn_(maize)___healthy; 10, Corn_(maize)___Northern_Leaf_Blight; 11, Grape___Black_rot; 12, Grape___Esca_(Black_Measles); 13, Grape___healthy; 14, Grape___Leaf_blight_(Isariopsis_Leaf_Spot); 15, Orange___Haunglongbing_(Citrus_greening); 16, Peach___Bacterial_spot; 17, Peach___healthy; 18, Pepper,_bell___Bacterial_spot; 19, Pepper,_bell___healthy; 20, Potato___Early_blight; 21, Potato___healthy; 22, Potato___Late_blight; 23, Raspberry___healthy; 24, Soybean___healthy; 25, Squash___Powdery_mildew; 26, Strawberry___healthy; 27, Strawberry___Leaf_scorch; 28, Tomato___Bacterial_spot; 29, Tomato___Early_blight; 30, Tomato___healthy; 31, Tomato___Late_blight; 32, Tomato___Leaf_Mold; 33, Tomato___Septoria_leaf_spot; 34, Tomato___Spider_mites Two-spotted_spider_mite; 35, Tomato___Target_Spot; 36, Tomato___Tomato_mosaic_virus; and 37, Tomato___Tomato_Yellow_Leaf_Curl_Virus.* Figure S3: complexity of images generated by feature visualization quantified by Shannon entropy*. Images generated by feature visualization were converted to grayscale and their Shannon entropy was quantified. Maximum 300 images were randomly sampled from each layer. Lines and areas indicate the average and standard deviation, respectively.* Figure S4: application of Grad-CAM targeting different layers of the CNN*.* Figure S5: application of explanation map targeting different layers of the CNN*.
