# Supplementary material for: How Convolutional Neural Networks Diagnose Plant Disease
Source: Plant Phenomics. 2019 Mar 26;2019:9237136. doi: 10.34133/2019/9237136 (PMC7706313; doi:10.34133/2019/9237136)
Supplement: Supplementary Materials — Figure S1: overview of the visualization methods introduced in this paper. An image of Cavalier King Charles Spaniel is passed to the CNNs that were trained with ImageNet dataset. CNN predicts the image as “Blenheim_splaniel” (A breed of Cavalier) by 95.4%. Example images generated by Visualizations I to IV are displayed. See Materials and Methods for details of respective methods. Figure S2: details of the confusion matrix described inFigure 1(d). Ratio of classified images is described in each cell. Ticks represent the labels of the PlantVillage dataset. Class names corresponding to each label are as follows: 0, Apple___Apple_scab; 1, Apple___Black_rot; 2, Apple___Cedar_apple_rust; 3, Apple___healthy; 4, Blueberry___healthy; 5, Cherry_(including_sour)___healthy; 6, Cherry_(including_sour)___Powdery_mildew; 7, Corn_(maize)___Cercospora_leaf_spot Gray_leaf_spot; 8, Corn_(maize)___Common_rust; 9, Corn_(maize)___healthy; 10, Corn_(maize)___Northern_Leaf_Blight; 11, Grape___Black_rot; 12, Grape___Esca_(Black_Measles); 13, Grape___healthy; 14, Grape___Leaf_blight_(Isariopsis_Leaf_Spot); 15, Orange___Haunglongbing_(Citrus_greening); 16, Peach___Bacterial_spot; 17, Peach___healthy; 18, Pepper,_bell___Bacterial_spot; 19, Pepper,_bell___healthy; 20, Potato___Early_blight; 21, Potato___healthy; 22, Potato___Late_blight; 23, Raspberry___healthy; 24, Soybean___healthy; 25, Squash___Powdery_mildew; 26, Strawberry___healthy; 27, Strawberry___Leaf_scorch; 28, Tomato___Bacterial_spot; 29, Tomato___Early_blight; 30, Tomato___healthy; 31, Tomato___Late_blight; 32, Tomato___Leaf_Mold; 33, Tomato___Septoria_leaf_spot; 34, Tomato___Spider_mites Two-spotted_spider_mite; 35, Tomato___Target_Spot; 36, Tomato___Tomato_mosaic_virus; and 37, Tomato___Tomato_Yellow_Leaf_Curl_Virus. Figure S3: complexity of images generated by feature visualization quantified by Shannon entropy. Images generated by feature visualization were converted to grayscale and their Shannon entropy was quantified. Maxi [file 9237136.f1.pptx]

## Slide 1
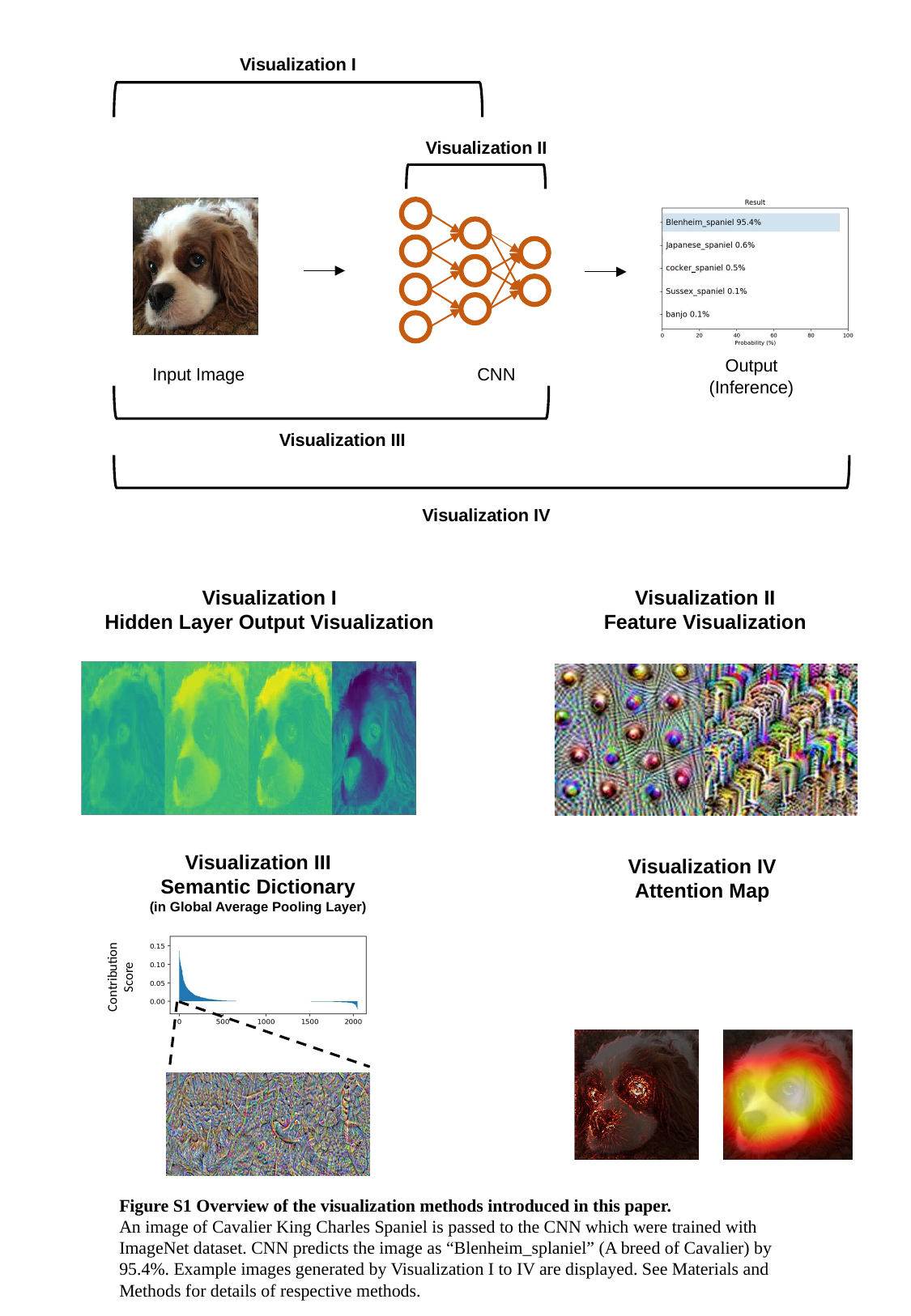

Visualization I
Visualization II
Input Image
Output
(Inference)
CNN
Visualization III
Visualization IV
Visualization I
Hidden Layer Output Visualization
Visualization II
Feature Visualization
Visualization III
Semantic Dictionary
(in Global Average Pooling Layer)
Visualization IV
Attention Map
Contribution
Score
Figure S1 Overview of the visualization methods introduced in this paper.
An image of Cavalier King Charles Spaniel is passed to the CNN which were trained with ImageNet dataset. CNN predicts the image as “Blenheim_splaniel” (A breed of Cavalier) by 95.4%. Example images generated by Visualization I to IV are displayed. See Materials and Methods for details of respective methods.

## Slide 2
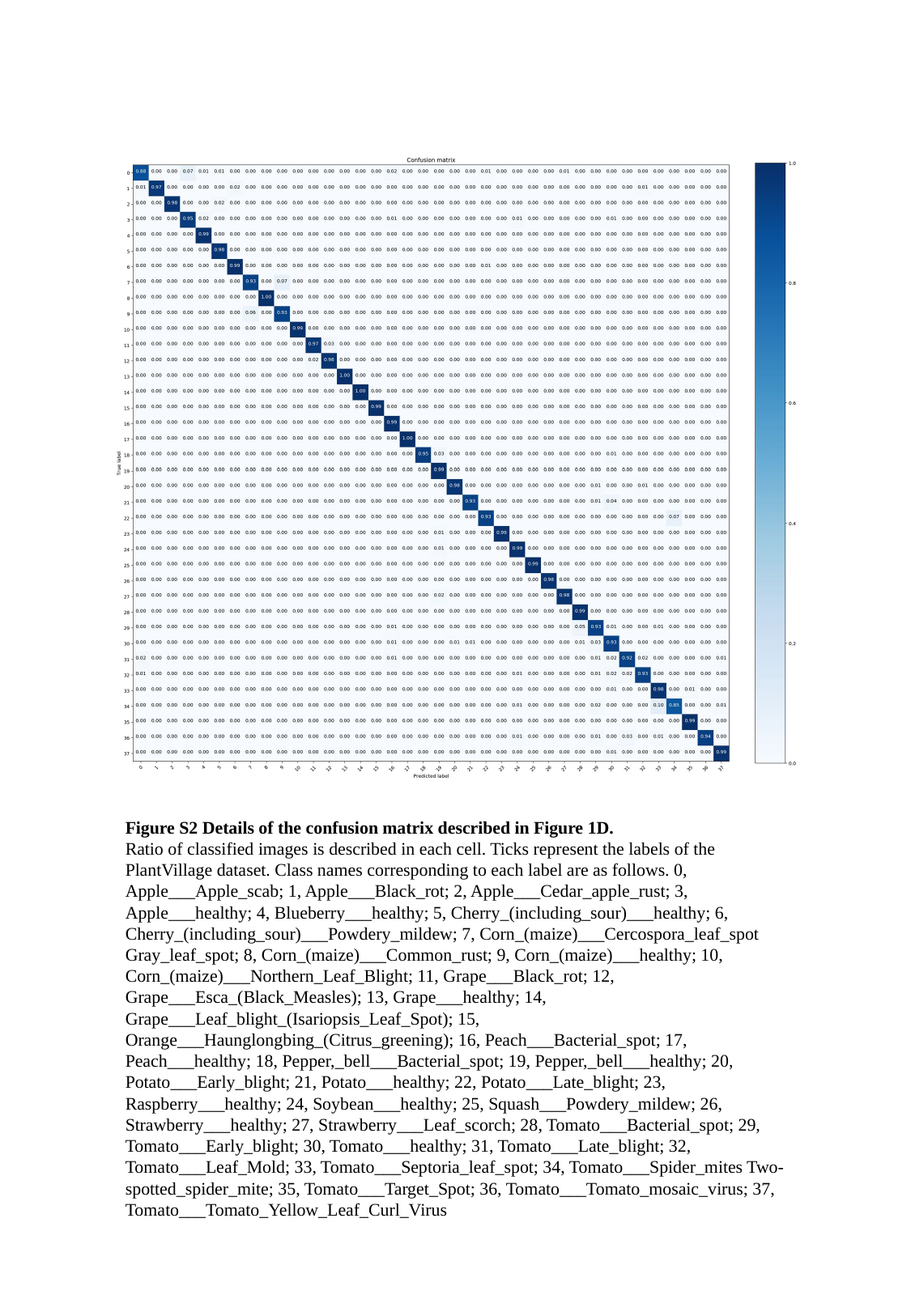

Figure S2 Details of the confusion matrix described in Figure 1D.
Ratio of classified images is described in each cell. Ticks represent the labels of the PlantVillage dataset. Class names corresponding to each label are as follows. 0, Apple___Apple_scab; 1, Apple___Black_rot; 2, Apple___Cedar_apple_rust; 3, Apple___healthy; 4, Blueberry___healthy; 5, Cherry_(including_sour)___healthy; 6, Cherry_(including_sour)___Powdery_mildew; 7, Corn_(maize)___Cercospora_leaf_spot Gray_leaf_spot; 8, Corn_(maize)___Common_rust; 9, Corn_(maize)___healthy; 10, Corn_(maize)___Northern_Leaf_Blight; 11, Grape___Black_rot; 12, Grape___Esca_(Black_Measles); 13, Grape___healthy; 14, Grape___Leaf_blight_(Isariopsis_Leaf_Spot); 15, Orange___Haunglongbing_(Citrus_greening); 16, Peach___Bacterial_spot; 17, Peach___healthy; 18, Pepper,_bell___Bacterial_spot; 19, Pepper,_bell___healthy; 20, Potato___Early_blight; 21, Potato___healthy; 22, Potato___Late_blight; 23, Raspberry___healthy; 24, Soybean___healthy; 25, Squash___Powdery_mildew; 26, Strawberry___healthy; 27, Strawberry___Leaf_scorch; 28, Tomato___Bacterial_spot; 29, Tomato___Early_blight; 30, Tomato___healthy; 31, Tomato___Late_blight; 32, Tomato___Leaf_Mold; 33, Tomato___Septoria_leaf_spot; 34, Tomato___Spider_mites Two-spotted_spider_mite; 35, Tomato___Target_Spot; 36, Tomato___Tomato_mosaic_virus; 37, Tomato___Tomato_Yellow_Leaf_Curl_Virus

## Slide 3
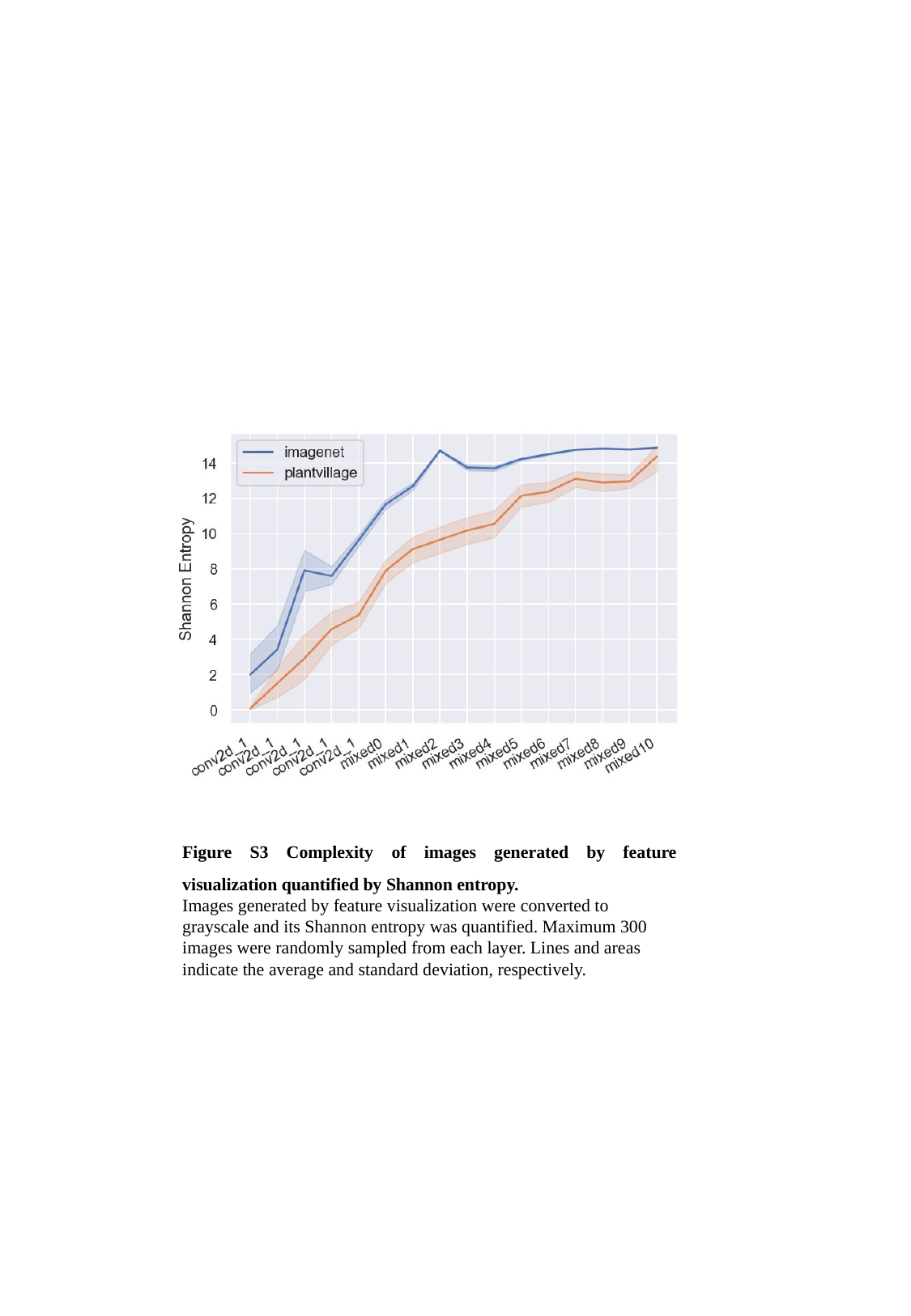

Figure S3 Complexity of images generated by feature visualization quantified by Shannon entropy.
Images generated by feature visualization were converted to grayscale and its Shannon entropy was quantified. Maximum 300 images were randomly sampled from each layer. Lines and areas indicate the average and standard deviation, respectively.

## Slide 4
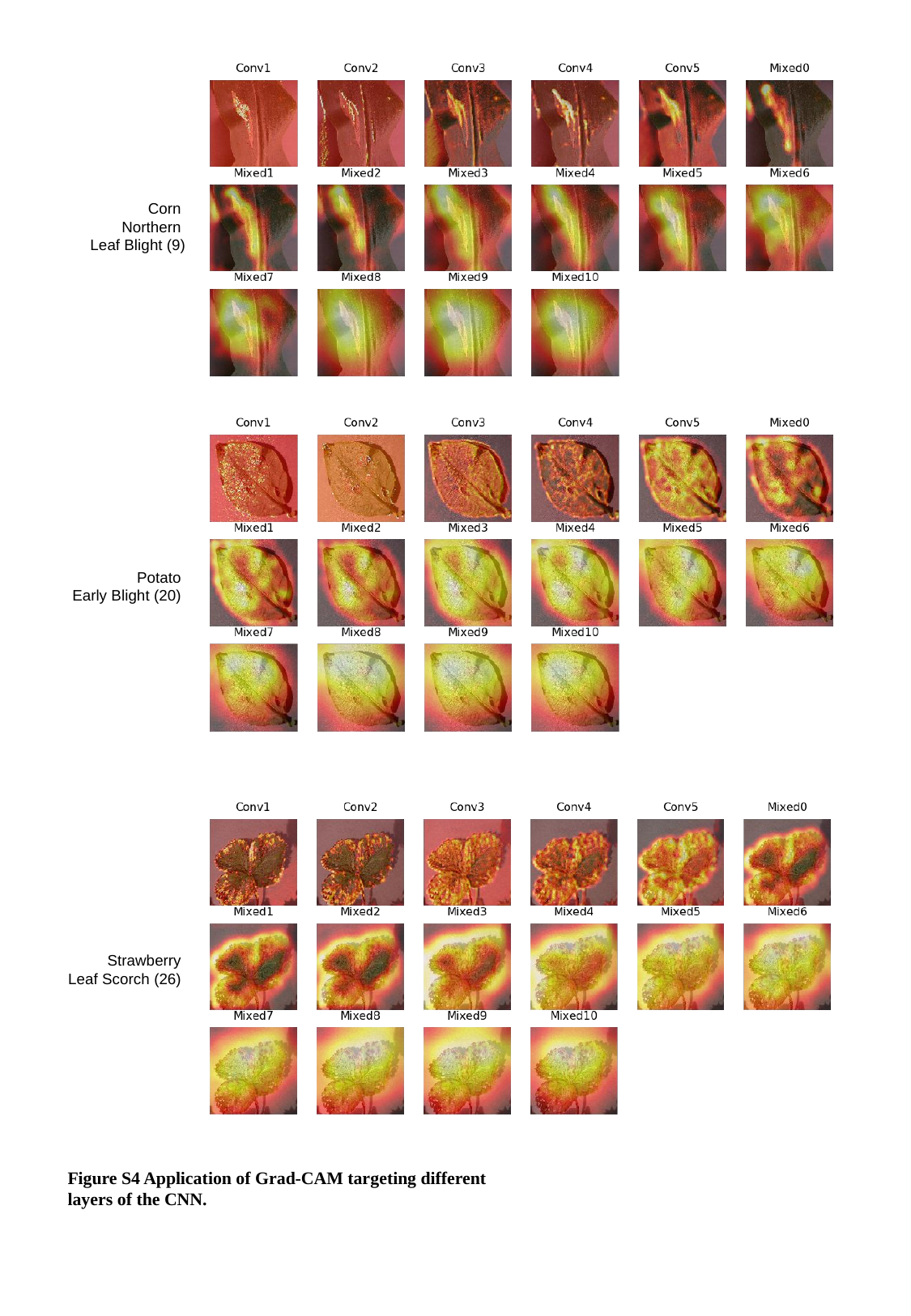

Corn
Northern
Leaf Blight (9)
Potato
Early Blight (20)
Strawberry
Leaf Scorch (26)
Figure S4 Application of Grad-CAM targeting different layers of the CNN.

## Slide 5
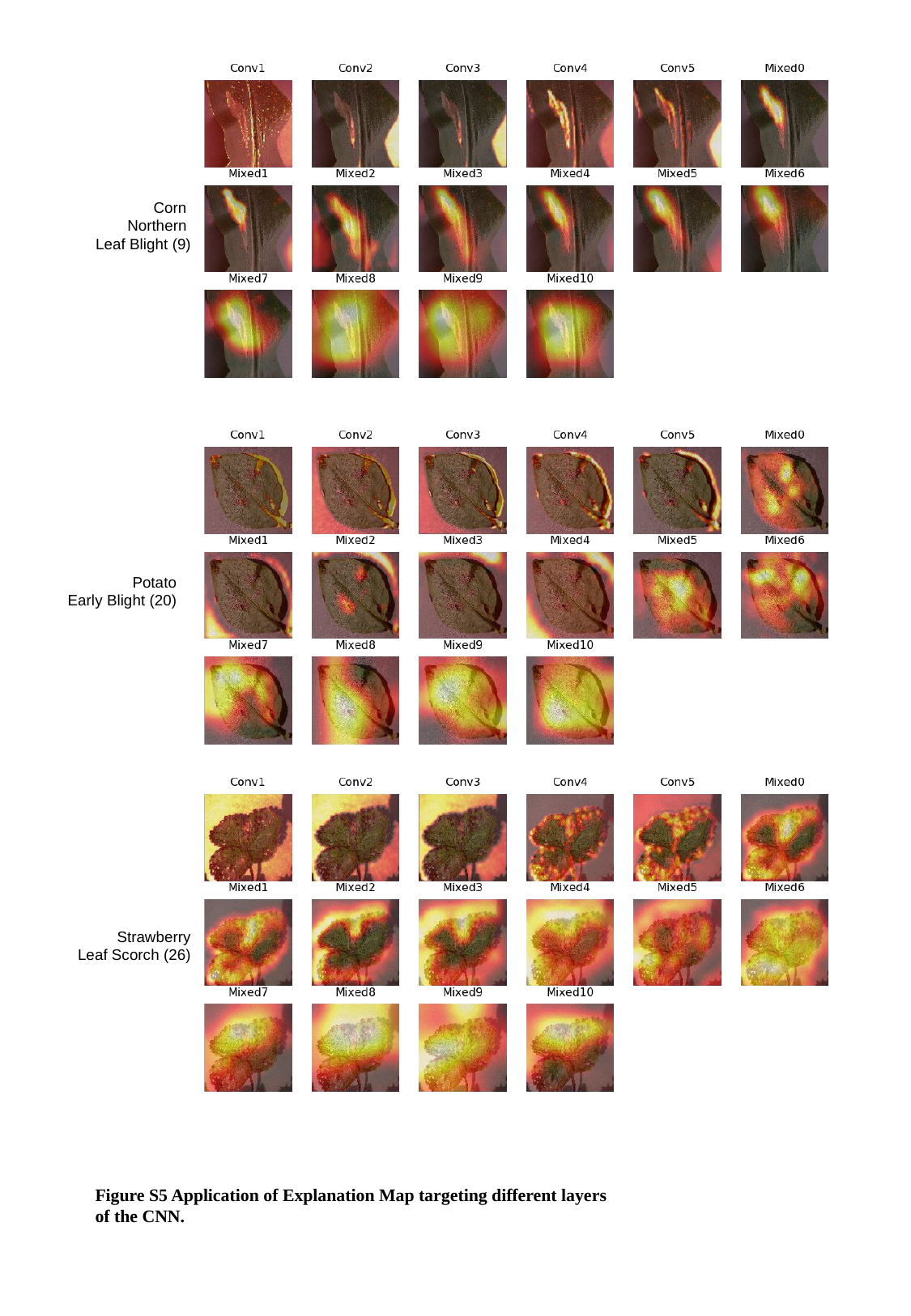

Corn
Northern
Leaf Blight (9)
Potato
Early Blight (20)
Strawberry
Leaf Scorch (26)
Figure S5 Application of Explanation Map targeting different layers of the CNN.
